# Identification of a GPR182-postive stem cell population that drives polyp progression in familial adenomatous polyposis

**DOI:** 10.7717/peerj.20704

**Published:** 2026-03-27

**Authors:** Ruoyu Wu, Yuhang Ling, Ying He, Linhua Yao, Qian Shi, Weiyun Shen, Xinbo Li, Yan Liu, Jingjing Li

**Affiliations:** 1 Department of Gastroenterology, First Affiliated Hospital of Huzhou University, Huzhou, Zhejiang, China; 2 School of Medicine, Huzhou University, Huzhou, Zhejiang, China; 3 Huzhou Key Laboratory of Translational Medicine, First Affiliated Hospital of Huzhou University, Huzhou, Zhejiang, China; 4 Central Laboratory, First Affiliated Hospital of Huzhou University, Huzhou, Zhejiang, China; 5 Department of General Practice, First Affiliated Hospital of Huzhou University, Huzhou, Zhejiang, China

**Keywords:** Single-cell sequencing, Familial adenomatous polyposis, GPR182, Machine learning, Biomarker

## Abstract

**Background:**

Familial adenomatous polyposis (FAP) is characterized by hundreds of colorectal adenomas that inevitably progress into carcinomas. This study focused on the heterogeneity during the polyposis progression to identify new targets and signatures for therapeutic development.

**Methods:**

An integrated analysis of single-cell sequencing (GSE109308) and bulk transcriptomic data (GSE79460, GSE88945, GSE94919, GSE106500, GSE109812, GSE153385, and GSE156172) of FAP patients from the Gene Expression Omnibus database was conducted. The heterogeneous features of epithelial cell clusters were described in terms of evolutionary trajectory, stemness, hypoxia, epithelial-mesenchymal transition (EMT), immune infiltration and metabolism. Three machine learning algorithms were applied to identify the key cell subset driving polyp heterogeneity, followed by functional validation with cell cycle and viability experiments. Cell-cell communication landscapes of this significant cell subset and its associations with prognosis and response to chemotherapy or immunotherapy were delineated.

**Results:**

Thirteen epithelial cell clusters were determined and further classified into four heterogeneous phenotypes. A specific cell population, G protein-coupled receptor 182 (GPR182)-positive polyp stem cells (GPR182^+^ PSCs), was identified as a crucial contributor to heterogeneity. The GPR182^+^ PSCs showed tumor-priming capacity in evolutionary trajectory analysis and exhibited extensive cell-cell communication with immune cells, especially M2 macrophages and T cells. Importantly, high abundance of GPR182^+^ PSCs correlated with poor prognosis and elevated expression of immune checkpoints (PD-L1 and CTLA-4) in colorectal cancer. GPR182^+^ PSCs could also predict the responses of colorectal cancer patients to certain drug treatments, such as rapamycin and midostaurin.

**Conclusion:**

Our findings map the epithelial heterogeneity in FAP and reveal that GPR182^+^ PSCs are crucial in driving heterogeneity and immune evasion. GPR182^+^ PSCs represent promising biomarkers for prognosis and drug responses, providing novel insights into FAP pathology and therapeutic development.

## Introduction

Familial adenomatous polyposis (FAP) is known as a hereditary colorectal disorder characterized by numerous polyps or adenomas (>100) that develop throughout the colorectum (Zaffaroni et al., 2024). It is an autosomal dominant syndrome caused by a germline mutation in the adenomatous polyposis coli (APC) gene located on chromosome 5q21, which progresses into colorectal cancer (CRC) without prompt prophylactic colectomy. FAP patients additionally exhibit serious extraintestinal manifestations, including duodenal, gastric, and small intestine adenomas, osteomas, nasopharyngeal angiofibromas, desmoid fibromatosis, and increased risks of brain, pancreas, liver, and adrenal gland tumors (Dinarvand et al., 2019). Current guidelines therefore recommend annual colonoscopic surveillance to determine the optimal timing and extent of colectomy. Unfortunately, surgery could not eliminate the lifelong risk of extracolonic tumors. Substantial investigations have been conducted to develop chemo-preventive agents that can temporize the need for surgery and attenuate extracolonic disorders; however, no ideal drug candidates have been found to date (Kemp Bohan et al., 2021; Lauricella et al., 2024). Thus, there is still an urgent need for further understanding of the pathological characteristics of FAP to develop novel, efficient, and safe treatment strategies.

Single-cell sequencing (SCS) has emerged as a powerful tool that enables the detailed dissection of complex tumor biology at the single-cell level. Based on genome-wide expression data from SCS, new cell populations with specific gene signatures can be identified, cell evolution trajectory can be determined, and cell-cell communication between different cell types can be elucidated (Lei et al., 2021; Lim, Lin & Navin, 2020). Another important application area of SCS is drug development, where SCS data enables a deeper understanding of the influence of drug agents and therapy on the immune-tumor microenvironment (TME). This, in turn, enables more precise targeting of tumors and helps uncover the mechanisms underlying drug response and resistance (Lim, Lin & Navin, 2020). For instance, in a study of basal cell carcinoma patients, single-cell ATAC sequencing data revealed that exhausted T cells were extremely expanded after anti-PD-1 therapy, highlighting that the PD-1 blockade affected both CD8^+^ and CD4^+^ T cells (Satpathy et al., 2019). Single-cell RNA sequencing has also advanced our knowledge of FAP and sporadic colorectal cancer (Becker et al., 2022; Li et al., 2020). One study of FAP patients uncovered that adjacent polyps likely initiated from the same tumor-prone cell, with precancerous adenomas already displaying metabolic reprogramming (Li et al., 2020). However, SCS-based studies on FAP remain limited, and the single-cell tumor biology of FAP is incompletely understood.

In this study, we conducted an integrated analysis of single-cell RNA sequencing and bulk transcriptomic data from FAP patients to explore epithelial heterogeneity, evolutionary trajectory, metabolic reprogramming and immune crosstalk. Using three machine-learning frameworks, we identified a distinct stem cell population, GPR182^+^ polyp stem-cells (GPR182^+^ PSCs), and comprehensively evaluated its roles in stem cell proliferation, prognosis, drug-response prediction, and interplay with immune cells.

## Materials and Methods

### Datasets used for analysis

The processed scRNA-seq data of FAP samples and normal tissues were obtained from the GEO database (GSE109308) and used for the analysis of tumor microenvironment. Bulk RNA-seq data (GSE88945, GSE94919, GSE106500, GSE153385, and GSE156172) and microarray data (GSE9689, GSE79460, and GSE109812) from GEO were downloaded. Additionally, FAP colon organoid datasets (GSE207398 and GSE109814) were used to validate the cell composition scores derived from bulk tissue and refine the drug sensitivity predictions by specifically assessing the epithelial cell response.

For RNA-seq datasets provided as raw counts, expression levels were converted into reads per kilobase of transcript per million mapped reads (RPKM) using a custom R script. For microarray datasets, probe identifiers were mapped to official gene symbols using the corresponding platform annotation files (*e.g*., GPL3408 for GSE9689). In cases where multiple probes mapped to the same gene, the probe with the maximum expression value was retained. The processed RNA-seq and microarray datasets were then merged into a single expression matrix based on common gene symbols. To mitigate batch effects arising from different platforms and studies, the combined matrix was log-transformed (log(x + 1)) and corrected using the ComBat function from the sva R package, with the dataset of origin specified as the batch variable and par.prior set to FALSE (Leek et al., 2012). The effectiveness of batch effect removal was confirmed by principal-component analysis (PCA). The final integrated dataset, along with its corresponding metadata, was used for all subsequent bulk tissue analyses.

### Single-cell transcriptome analysis

Single-cell transcriptome data were imported into Seurat (v2.3.0) R package for quality control and downstream analysis. Initial quality-control removed cells expressing <300 genes, genes detected in <3 cells, and cells whose mitochondrial transcript fraction exceeded 20% or whose haemoglobin transcript fraction exceeded 0.1%. The filtered data were normalized using the NormalizeData function, and highly variable features were identified using the FindVariableFeatures function. The data were then scaled, and dimensionality reduction was performed using PCA. The top 10 principal components (PCs) were used for t-SNE and UMAP visualization, as well as for graph-based clustering (FindNeighbors, dims 1:10; FindClusters, resolution = 0.5). Automated cell type annotation was performed using the SingleR R package in conjunction with the celldex::HumanPrimaryCellAtlasData reference dataset (Aran et al., 2019). To further investigate epithelial cell heterogeneity, cells annotated as “Epithelial_cells” were re-processed through the standard Seurat workflow: NormalizeData, FindVariableFeatures, ScaleData, PCA, t-SNE/UMAP (30 PCs), and FindClusters (resolution = 0.5). Marker genes for each epithelial sub-cluster were identified using the FindAllMarkers function (only.pos = TRUE, min.pct = 0.25, and logfc.threshold = 0.1).

To infer the developmental trajectory of epithelial cell subsets, the Monocle(v2) R package was employed (Newman et al., 2015). A CellDataSet object was created from the raw count matrix of epithelial cell subset. After estimation of size factor and dispersion, genes detected in ≥10 cells were retained. The top 800 differentially expressed genes across clusters (q-value < 0.01) were used to order the cells along pseudotime. Dimensionality was reduced using the “DDRTree” method, and cells were ordered along the inferred trajectory.

### Bulk RNA-seq data analysis

Bulk RNA-seq data from seven datasets were merged, and batch effects were removed with the ComBat function of R package SVA (Leek et al., 2012); successful removal of batch effects was verified by PCA. “ConsensusClusterPlus” was used to perform cluster analysis, which identified 13 epithelial cell subset-related subtypes. The expression matrix of DEGs was used as the input. Clustering was performed using the Partitioning Around Medoids (PAM) algorithm with ‘pearson’ distance, iterating k = 2–6 clusters across 500 resampling runs (pItem = 0.8, pFeature = 1). The optimal cluster number was determined based on the consensus matrix and the cumulative-distribution-function (CDF) plots. Relative abundance of 22 immune-cell types in the tumor microenvironment was estimated with CIBERSORT algorithm (1,000 permutations, quantile normalisation = TRUE). For this analysis, normalized bulk expression data served as the input mixture file. The landmark LM22 gene signature matrix was used as the reference.

To assess enrichment of selected pathways or cell signatures in bulk and organoid datasets, Gene Set Variation Analysis (GSVA) was performed using the GSVA R package (Hänzelmann, Castelo & Guinney, 2013). The PSC signature and activities of 160 immune-related pathways were evaluated by the single-sample gene set enrichment Analysis (ssGSEA) method on a non-log-transformed expression matrix. To validate the presence of the PSC signature, marker genes for cluster 5 (logfc.threshold = 0.25, min.pct = 0.25, only.pos = TRUE) were used as a custom gene set. To estimate the proportion of the scRNA-seq-defined epithelial cluster 5 in bulk tumor samples, we utilized the BisqueRNA R package (Jew et al., 2020). A reference single-cell expression profile was constructed from our annotated epithelial scRNA-seq data, and the integrated bulk RNA-seq dataset served as the mixture. The ReferenceBasedDecomposition function was executed with cell.type = “cellType”, subject.name = “SubjectName”, and use.overlap = FALSE. The estimated proportions of cluster 5 were then compared across different patient groups using the Wilcoxon rank-sum test.

### Machine learning algorithms employed for analysis

Support vector machine (SVM), extreme gradient boosting (XGBoost), and random forest (RF) were applied to the batch-corrected bulk RNA-seq data to build classification models. One hundred and ninety-five samples were split into a training set (*n* = 136, 70%) and an independent test set (*n* = 59, 30%). SVM model with a radial basis function kernel was trained using the e1071 package. eXtreme Gradient Boosting (XGBoost) was constructed using the xgboost R package (objective, binary:logistic for binary classification). Key hyperparameters were tuned *via* cross-validation (nrounds = 50, maximum tree depth = 100). Feature importance scores were calculated to identify the most influential predictors. The RF model was built using the randomForestSRC package (ntree = 800). Feature importance was assessed, and the model’s error rate was evaluated using 5-fold cross-validation (rfcv function). The final set of diagnostic genes was determined by the intersection of features selected by all three models. Least Absolute Shrinkage and Selection Operator (LASSO) logistic regression by the glmnet package was used to reduce the number of candidate features *via* 10-fold cross-validation (cv.glmnet function) and the final features were selected based on the lambda.min criterion. A risk score (signature) was calculated for each sample by summing the expression values of the final genes weighted by their LASSO regression coefficients. Model performance was evaluated by area under the receiver-operating-characteristic (ROC) curves.

### Cell communication analysis

Cell-to-cell communication was ascertained by R package CellChat (v1.5.0) through evaluating ligand-receptor expression pairs as previously reported (Jin et al., 2021). The interactions between different cell types were examined and a gene-expression threshold of 0.2 was used. To enable a systematic analysis of cell-to-cell communication molecules, we also performed analysis using CellPhoneDB software (Efremova et al., 2020). Membrane-bound and peripheral proteins of different cell types were annotated. Statistical significance (*p* value < 0.05) was determined according to the interaction scores generated by the normalized cell matrix with Seurat. Using cell-state labels and the TPM expression profile of GPR182^+^ PSC cells and immune cells as input, a three-dimensional pseudo-space reconstruction was performed with CSOmap (https://doi.org/10.24433/CO.8641073.v1).

### Cell culture and transfection

The human normal colon epithelial cell line, HCoEpiC, was obtained from Type Culture Collection of the Chinese Academy of Sciences (Shanghai, China). HCoEpiC cells were cultured in DMEM medium supplemented by 10% fetal bovine serum (Gibco, Waltham, MA, USA). Human GPR182 CDS was cloned into the pcDNA3.1-3×Flag-C plasmid to construct the overexpression vector. pcDNA3.1-GPR182-3×Flag-C plasmid or empty vector was subsequently transfected into cultured HCoEpiC cells using EzTrans reagent (Shanghai Life iLab Bio-Technology, Shanghai, China) according to the manufacturer’s instructions.

### Immunofluorescence

The precancerous colon mucosa tissues were retrospectively collected from CRC patients who received surgical resection at the First Affiliated Hospital of Huzhou University between Jan 2018 and Oct 2019. This study was conducted under the approval of the Ethics Committee of the First People’s Hospital of Huzhou (No. 2020KYLL055). Written informed consent was obtained from all patients. Tissue sectioning and IF staining of paraffin-embedded specimens were performed using standard protocols. Firstly, antigen retrieval was performed for the tissue sections (4 μm thick), after which slides were incubated with primary antibodies against GPR182 (1:100, Cat# 199177; Abcam, Cambridge, UK), LGR5 (1:50, Cat# A10545; Abclonal, Wuhan, China), CD206 (1:50, Cat# 60143-1-Ig; Proteintech, Wuhan, China), CD11b (1:100, Cat# 66519-1-Ig; Proteintech, Wuhan, China), or CD8a (1:100, Cat# 66868-1-Ig; Proteintech, Wuhan China). This was followed by incubation with appropriate HRP-conjugated secondary antibodies for 50 min, and then with fluorescently labeled tyramides (iF488-Tyramide, iF555-Tyramide, iF647-Tyramide) for 10 min. Sections were mounted with DAPI-containing medium (Invitrogen, Carlsbad, CA, USA). Images were captured using a 3D-HISTECH scanner (Pannoramic MIDI).

### Cell viability assay

Cell viability of HCoEpiC cells was measured using Cell Counting Kit-8 (CCK-8) (Beyotime, Shanghai, China). Cells were seeded in 96-well plates (2,000 cells/well) in triplicate. At the indicated time points, 10 μL CCK-8 solution was added to cultured cells in each well. The plates were subsequently incubated at 37 °C for 90 min. The optical density (OD) values were measured at 450 nm by SpectraMax 190 (Molecular Devices, San Jose, CA, USA).

### Cell cycle analysis

Cells were fixed in 70% ethanol and stained with propidium iodide (PI). Cell cycle profiles were analysed on a BD FACSCanto flow cytometer with FlowJo V10.

### Cancer stem cell sphere formation assay

Cells were harvested from normal culture 24 h after transfection and seeded into ultra-low attachment 6-well plates (Corning, Corning, NY, USA, 5,000 cells/well). These cells were incubated with serum-free DMEM/F12 medium containing 10 ng/mL of hrbFGF (Peprotech, Cranbury, NJ, USA) and 20 ng/mL of hrEGF (Peprotech, Cranbury, NJ, USA), 1% Insulin-Transferrin-Selenium (Gibco, Waltham, MA, USA), 2% B27 (Gibco, Waltham, MA, USA), and 100 U/mL penicillin and streptomycin solution at 37 °C with 5% CO_2_. After incubation for 6 days, the spheres that exceeded 20 cells were recorded.

### Survival and correlation analysis using TCGA data

COAD data were downloaded from The Cancer Genome Atlas (TCGA) through the Genomic Data Commons Data Portal. GPR182^+^ PSC scores were determined using the GSVA method as previously described. Survival curves were depicted using the survfit package with a cut-off value of mean PSC score. The correlation between GPR182^+^ PSC scores and PD-L1 and CTLA-4 expression levels was analyzed with Spearman’s test.

### Drug response prediction

The potential response to various therapeutic agents was predicted based on the risk scores. By using pRRophetic package (Geeleher, Cox & Huang, 2014), the half-maximal inhibitory concentrations (IC50s) for drugs were predicted from the Cancer Genome Project (CGP) 2016 genomics of drug sensitivity database. The prediction was performed with batchCorrect set to “eb” (Empirical Bayes) and tissueType specified as “urogenital_system” or “all” where appropriate. Additional chemosensitivity estimates were obtained with oncoPredict using the Genomics of Drug Sensitivity (GDSC) v2 database (Maeser, Gruener & Huang, 2021). The calcPhenotype function was run with batchCorrect = “eb” and powerTransformPhenotype = TRUE. The correlation between the calculated risk scores and the predicted IC50 values for each drug was determined using Pearson correlation. Significant associations were visualized using heatmaps generated with the ComplexHeatmap package.

### Statistical analysis

All statistical analyses were performed in R v4.2.0 and GraphPad Prism (v 9.0). Each *in vitro* experiment was independently repeated at least twice with triplicate replicates. Data were presented as mean ± SEM. Survival differences were evaluated with the log-rank test. Two-sided *p*-values < 0.05 were considered statistically significant.

## Results

### Epithelial heterogeneity landscape in FAP

**Figure 1:**
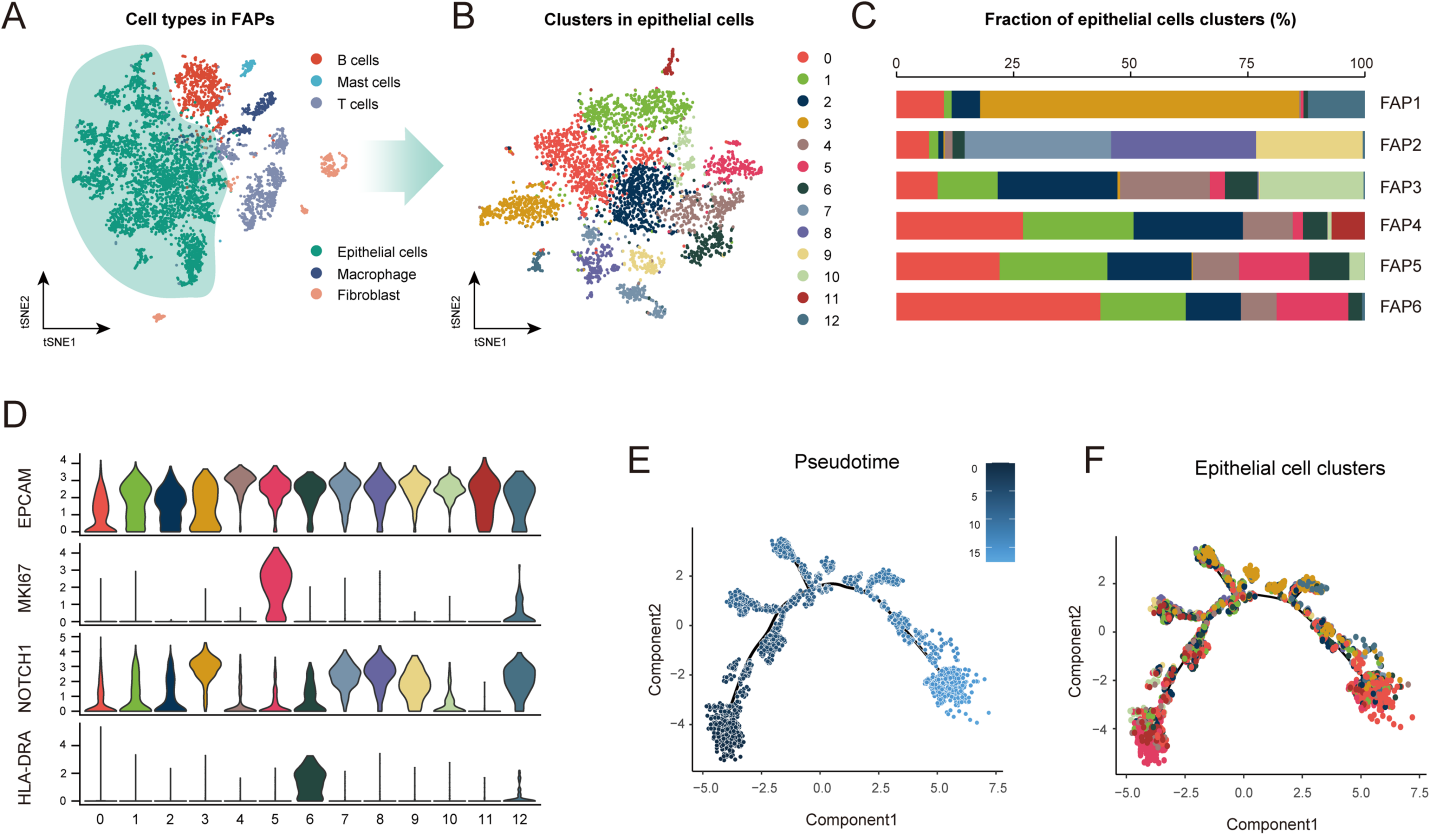
Epithelial heterogeneity landscape in FAP.

To comprehensively understand the diverse roles of epithelial cells in FAP development, we analyzed the single-cell sequencing data from six FAP patients from the GEO dataset (GSE109308). After data filtering and t-SNE analysis, 8,086 cells were classified into six distinct clusters, including epithelial cells, B cells, mast cells, T cells, macrophages, and fibroblasts (Figs. 1A, S1A). Among these clusters, epithelial cells were the majority, comprising 64.88% of all classified cells (Fig. S1B). Subsequently, epithelial cells were subjected to ClusterTree analysis (the optimal RNN, 0.5), which identified 13 clusters with distinct gene signatures (Figs. 1B, S1C). As shown in Fig. 1C, the proportions of the 13 clusters varied markedly amongst the six FAP patients, suggesting epithelial heterogeneity as well as varying gene expression patterns across these patients. Furthermore, we selected several markers related to tumor progression to profile heterogeneity among the epithelial clusters. All clusters were found to express EPCAM (epithelial cell marker), confirming their epithelial cell identity. Cluster 5 exhibited the highest expression of Ki-67 (proliferation marker), whereas Cluster 6 showed the highest expression of HLA-DRA (immune marker), indicating differences in proliferation activity and mucosal immune responses. Compared with the other subsets, Cluster 3, 7, 8, 9, and 12 showed elevated expression of Notch 1, a cell differentiation marker, suggesting a higher differentiation capacity (Fig. 1D). To explore the evolutionary trajectories of the 13 clusters, pseudotime analysis was performed using the Monocle package based on transcriptional levels of default developmental genes. Importantly, cells in Cluster 5 aggregated at the beginning of the evolutionary tree, indicating this cluster may be the driving population in FAP development. The majority of Cluster 0 appeared at the end of the trajectory, suggesting it represents a teminal cell state (Figs. 1E, 1F). Furthermore, we displayed the top 100 differentially expressed genes along the evolutionary tree in a heatmap, which revealed six distinct gene expression patterns (Fig. S2A). Multiple genes within these patterns have been implicated in tumor progression. Such genes include: apolipoprotein B mRNA editing enzyme catalytic subunit 3B (*APOBEC3B*) (Burns, Temiz & Harris, 2013), immunoglobulin lambda-like polypeptide 5 (*IGLL5*) (White et al., 2018), cytochrome P450 family 3 subfamily A member 4 (*CYP3A4*) (van Eijk et al., 2019), and C-C motif chemokine ligand 20 (*CCL20*) (Kadomoto, Izumi & Mizokami, 2020). For example, APOBEC3B has been reported to drive mutation and carcinogenesis (Burns, Temiz & Harris, 2013), which is consistent with its high expression at the beginning of the evolutionary trajectory. IGLL5, a novel recurrence marker, was found to be gradually up-regulated in the evolutionary process, indicating its involvement in migration and invasion in advanced-stage FAP. Subsequent CytoTrace analysis elucidated the differentiation capacities of these epithelial clusters. Cluster 5 at the root of the tree had the highest CytoTrace scores, suggesting it may play a role in the origin of FAP tumors (Fig. S2B). Taken together, these data demonstrate that epithelial cells display considerable heterogeneity and are likely to play distinct roles during FAP onset and progression.

### Stratification of FAP patients upon epithelial cell signatures from single-cell analysis

**Figure 2:**
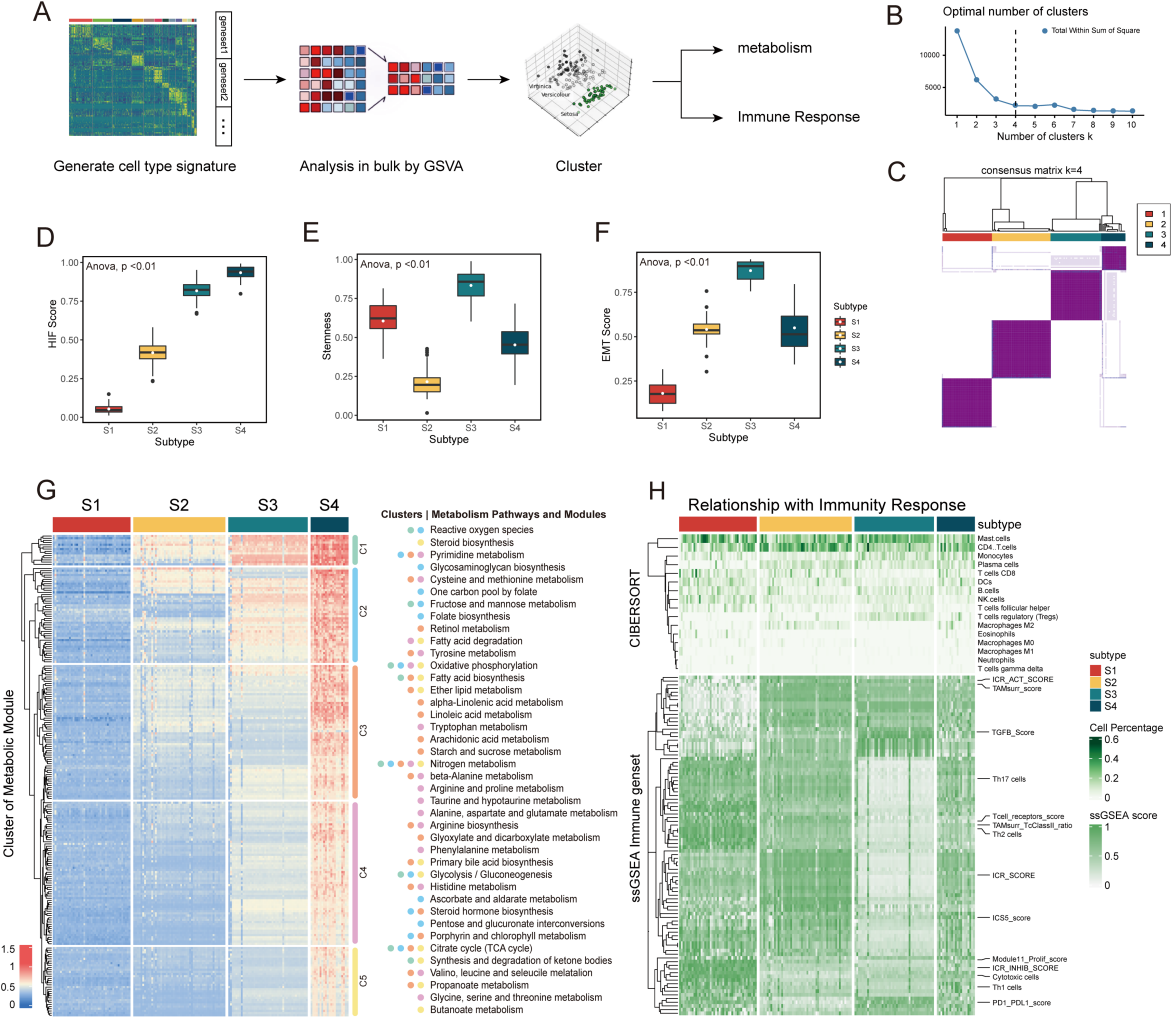
Grouping of FAP patients upon epithelial cell signatures from single-cell analysis.

Building upon the identification of distinct epithelial cell signatures and their potential roles in FAP development from the single-cell analysis (Table S1), we sought to translate these findings into a clinically relevant stratification of FAP patients. We conducted patient clustering for seven FAP transcriptome datasets (GSE79460, GSE88945, GSE94919, GSE106500, GSE109812, GSE153385, and GSE156172) to stratify patients, as well as characterize their disease states. The GSVA algorithm was used to score epithelial cell subsets of each patient according to gene tags shown in Table S1 and the optimal number of clusters was determined to be four by the Calinsky method (Figs. 2A, 2B). Other relevant clustering results were analyzed *via* NbClust and PAM K-mean algorithms and were displayed in Figs. S3A–S3D. A total of 195 patients were divided into four subpopulations (S1–S4) *via* Consensus Clustering (Fig. 2C). We next focused on three key tumor hallmarks, hypoxia, stemness, and EMT, across these four subpopulations. Hypoxia scores were assessed using an established hypoxia metagene signature (Buffa et al., 2010). Stemness and EMT scores were assessed using machine learning methods (OCLR for stemness, MLR for EMT) as previously reported (George et al., 2017; Malta et al., 2018). Among the four subpopulations, S1 exhibited low hypoxia and EMT scores but a relatively high stemness score. This suggests tissues from S1 patients retain features of normal mucosa and likely represent an early stage in the polyp-adenoma-carcinoma sequence (Figs. 2D–2F). By contrast, S3 had the highest stemness and EMT scores, indicating that subpopulation S3 exhibits more aggressive phenotypes than S1 and has a greater potential to progress to advanced adenoma and carcinoma. The profiles of S2 and S4 were more heterogeneous across these hallmarks compared to S1 and S3, which implies that S2 and S4 may represent intermediate disease stages. Given the importance of metabolism and immunity in tumor progression, we further analyzed these features in each subpopulation. The expression of metabolism-related genes and enriched pathways were displayed in a clustered heatmap with five modules, C1–C5 (Fig. 2G). Notably, S1 showed low expression of most metabolic genes and reduced pathway activity, whereas S4 showed the opposite pattern, indicating metabolism differences between early and late disease stages. The low gene expression in S1 likely correlates with the tumor-initiating stage, before substantial metabolism reprogramming has occurred. This is consistent with the hallmark analysis. It is well-known that tumor progression is characterized by an increasing energy demand, which leads to metabolic reprogramming. This reprogramming involves alterations in gluconeogenesis, glutathione metabolism, and lipogenesis (Fedele et al., 2022). We observed increased expression of genes involved in glycolysis, pyrimidine metabolism, and oxidative phosphorylation from S1 to S4. Next, we evaluated the composition of infiltrating immune cells in each subpopulation using the CIBERSORT algorithm and outlined the immune response map by conducting single-sample gene set enrichment (ssGSEA) analysis on 160 immune expression signatures as previously reported (Thorsson et al., 2018) (Fig. 2H). As shown in the ESTIMATE analysis, S3 had the least enrichment of stromal cells and immune cells compared to S1, S2, and S4 (Figs. S3E–S3G). All four subpopulations showed rich infiltration of mast cells and CD4^+^ T cells, ranging from 0.2% to 0.6% across samples. Of note, immune constant of rejection (ICR) scores indicated active immune responses in S1, S2, and S4, whereas S3 exhibited a non-active immune state. Furthermore, S1 samples showed low enrichment of TGF-β signaling. Since TGF-β is often elevated in CRC and promotes carcinogenesis by aiding IL-22-producing CD4^+^ T cells (Perez et al., 2020), it is reasonable that S1 samples may primarily consist of hyperplastic polyps or low-grade adenomas. Moreover, all subpopulations except S2 showed moderate to high expression of PD-1/PD-L1 signatures, suggesting different responses to anti-PD-1 immunotherapy. The patient stratification based on epithelial cell signatures reveals distinct biological phenotypes across FAP progression, ranging from early-stage characteristics to more aggressive, late-stage disease. This analysis of tumor hallmarks, metabolism, and immune infiltration provides a foundational understanding of FAP heterogeneity. Crucially, the specific epithelial cell signatures that determine the stratification warrant further investigation.

### Identification of GPR182^+^ PSCs

**Figure 3:**
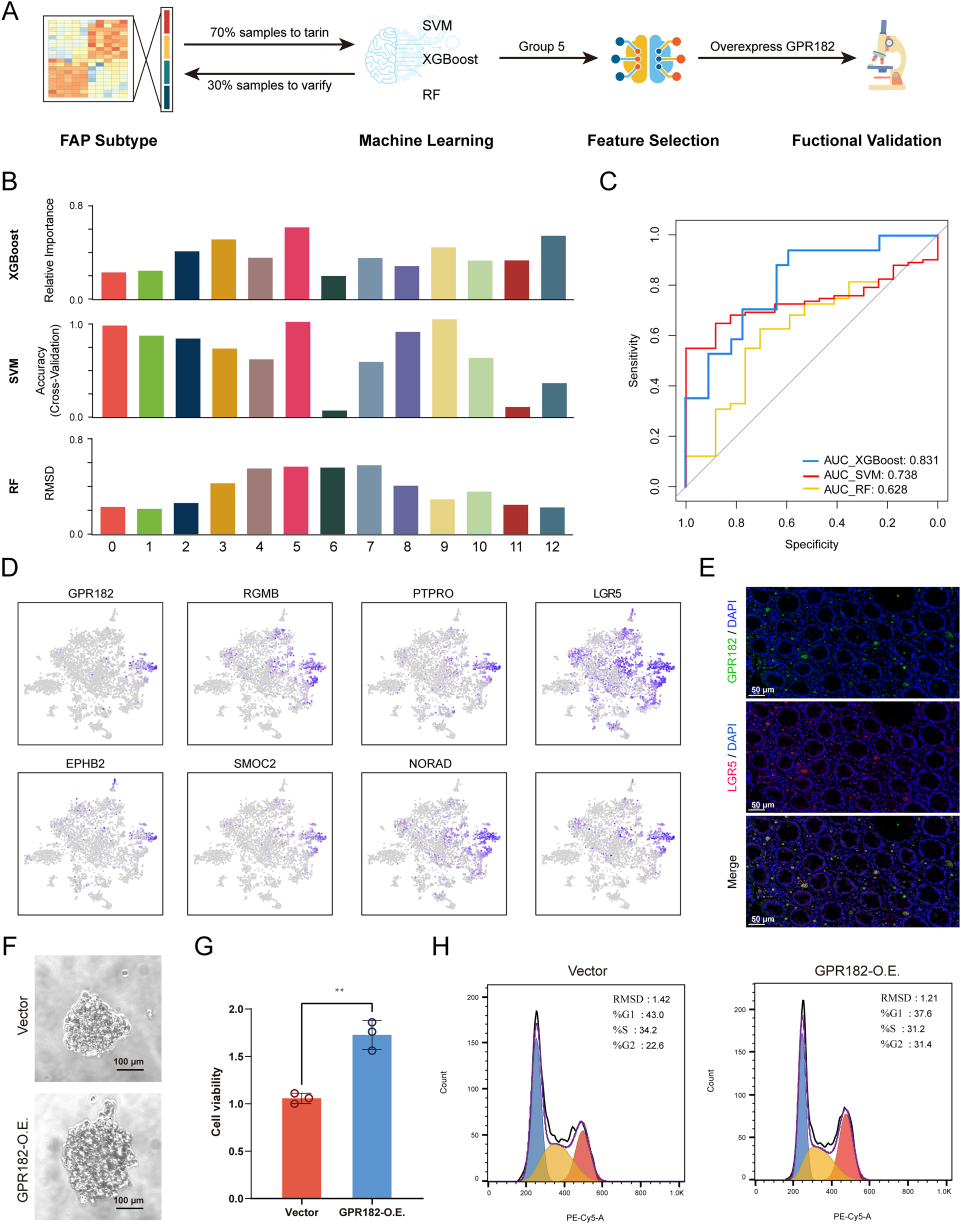
Identification of GPR182^+^ PSCs. GPR182 in promoting epithelial stem cell proliferation.

Motivated by the observed multiplex phenotypes across epithelial-signature-associated FAP subpopulations and the need to pinpoint key drivers of FAP progression, we employed machine learning approaches to identify critical discriminatory factors. Three algorithms, SVM, XGBoost, and RF, were used to select features and identify the most critical factors that affect stratification, which was validated by *in vitro* experiments (Fig. 3A). A total of 70% of samples (136 out of 195 samples) were used during the machine learning process and the signature of cluster 5 was found to be the most significant contributor in all three algorithms. Validation using the remaining 30% of samples (59 out of 195 samples) verified that the specificity and sensitivity were reliable and reproducible (Figs. 3B, 3C). The error scores of all three algorithms as well as the top 10 enriched pathways from the 13 epithelial clusters were displayed (Figs. S4A–S4D). Analysis of Cluster 5 revealed eight genes expressed in all FAP epithelial cells: *GPR182*, repulsive guidance molecule BMP co-receptor B (*RGMB*), protein tyrosine phosphatase receptor type O (*PTPRO*), leucine-rich repeat-containing G-protein-coupled receptor 5 (*LGR5*), EPH receptor B2 (*EPHB2*), SPARC-related modular calcium binding protein 2 (*SMOC2*), non-coding RNA activated by DNA damage (*NORAD*), and ribosomal protein L13 (*RPL13*). Among them, four genes are considered intestinal stem cell markers, namely *PTPRO* (Li et al., 2022), *LGR5*, *EPHB2* (Merlos-Suárez et al., 2011), and *SMOC2* (Jang et al., 2020), supporting potential stemness of Cluster 5 and its putative tumor-priming role in the evolutionary tree. We confirmed that GPR182 was significantly enriched in Cluster 5. Immunofluorescence studies further validated the co-expression of GPR182 with LGR5 at the base of the intestinal crypt (Figs. 3D, 3E). Based on these findings, we designated these cells as GPR182^+^ PSCs. *In vitro* culture of CSCs using HCoEpiC cells demonstrated that GPR182 overexpression contributed to the formation and growth of CSC colonies (Figs. 3F, 3G). Consistently, GPR182 overexpression was also found to accelerate the G1 to G2 phase transition in the cell cycle, indicating the role of GPR182 in promoting epithelial stem cell proliferation (Fig. 3H).

### The interplay between GPR182^+^ PSCs and immune cells

**Figure 4:**
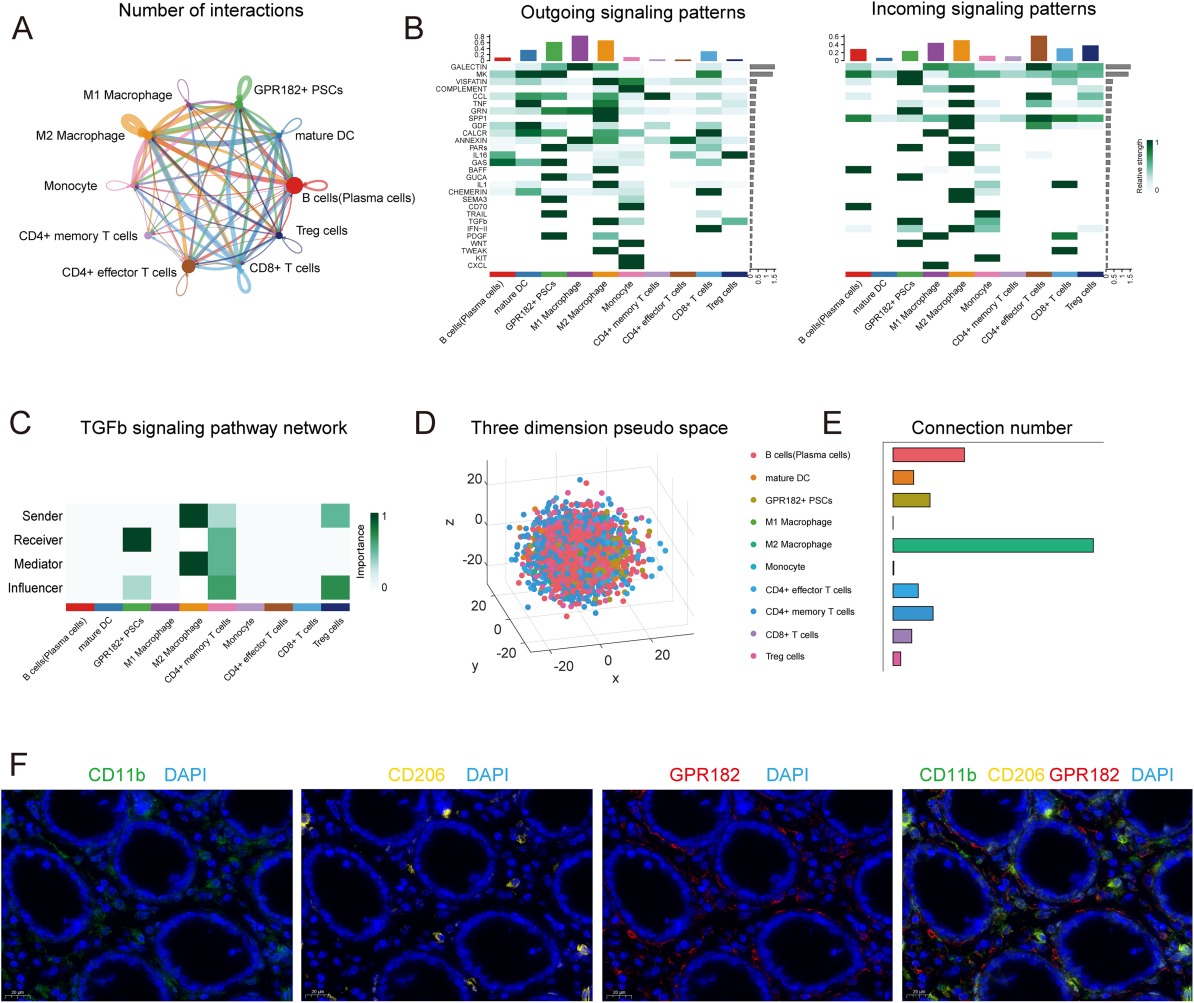
The interplay between GPR182^+^ PSCs and immune cells.

To clarify the role of GPR182^+^ PSCs, we analyzed intercellular communications among six cell types, namely epithelial cells, macrophages, T cells, B cells, mast cells, fibroblasts, and GPR182^+^ PSCs, using CellChat (Jin et al., 2021). Our data suggest extensive interactions between epithelial and immune cells (Fig. S5). Specifically, GPR182^+^ PSCs showed a high propensity to interact with multiple types of immune populations, such as M2 macrophages, B cells, and CD4^+^ T cells (Fig. 4A). To further explore how these cell populations coordinate in detailed signaling, we utilized CellphoneDB package in R to map outgoing and incoming signals. Amongst the 10 cell types, a plethora of ligand-receptor pairs were detected, which were further categorized into 28 pathways including: TGF-β, GAS, TRAIL, WNT, PDGF, GRN, MK, and SEMA3 signaling pathways (Fig. 4B). Of note, more than 15 significant ligand-receptor pairs involved GPR182^+^ PSCs, with M2 macrophages providing the most of interacting ligands and receptors, highlighting the pivotal regulatory role of M2 macrophages on GPR182^+^ PSCs. Interestingly, both paracrine and autocrine signaling were observed. For example, the PDGF signaling from GPR182^+^ PSCs to M2 macrophages and CD8^+^ T cells demonstrated a typical paracrine pattern. Similar paracrine patterns were also found between M2 macrophages and GPR182^+^ PSCs in GAS, SEMA3, TGF-β, and GRN signaling pathways. By contrast, GUCA, PARS, and MK signals represented autocrine pathways, with GPR182^+^ PSCs acting as the signal sender and receiver simultaneously. These findings indicate the complex and precise communication between GPR182^+^ PSCs and immune cells in the TME. Network centrality analysis revealed M2 macrophages as the dominent source of ligands and the key mediator of TGF-β signaling (Fig. 4C). Owing to the importance of TGF-β in CRC carcinogenesis (Perez et al., 2020), this interplay between GPR182^+^ PSCs and M2 macrophages might play a role in FAP progression.

Next, spatial reconstruction by CSOmap positioned M2 macrophages in close proximity to GPR182^+^ PSCs within a pseudo three-dimensional structure (Fig. 4D). In addition, M2 macrophages also exhibited the highest number of connections, suggesting their active cell-cell communication with other cell types in FAP tissues (Fig. 4E). Immunofluorescence images confirmed that GPR182^+^ cells were closely juxtaposed to CD11b^+^/CD206^+^ M2 macrophages (Fig. 4F) and resided within a short distance of CD8a^+^ T cells (Fig. S5B), indicating their interaction with M2 macrophages and CD8a^+^ T cells. The cell densities at the cross-section of Z = −20, −10, 0, 10, and 20 were displayed in Fig. S5C. Cell compactness of the M2 macrophages scored the highest out of 10 cell types analyzed, illuminating their role as a central hub in these 3D structures (Fig. S5D) (Ren et al., 2020). Consistent with the result in Figs. 4E, 4F, M2 macrophages initiated the highest number of statistically significant cell-cell signals (Fig. S5E).

### Predictive value of GPR182^+^ PSCs in prognosis, immunotherapy, and drug response

**Figure 5:**
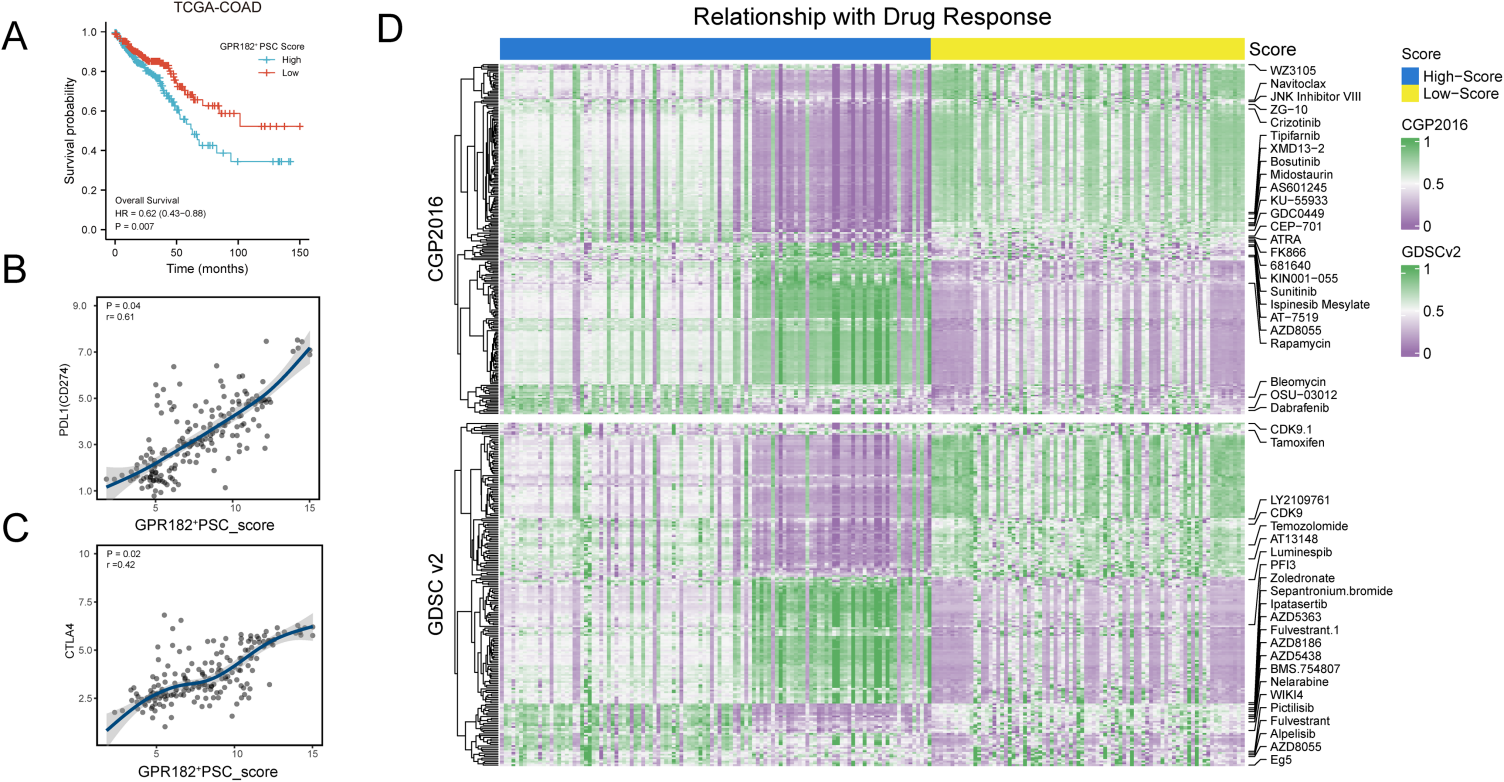
Predictive value of GPR182^+^ PSCs in prognosis, immunotherapy, and drug response.

Finally, we investigated the predictive value of GPR182^+^ PSCs in prognosis, immunotherapy, and drug responses for CRC and FAP patients. CRC patients from the TCGA database were scored according to GPR182^+^ PSC signatures by GSVA algorithm as mentioned above and then stratified into two groups. Kaplan-Meier curves illustrated patients with low GPR182^+^ PSC scores had significantly better overall survival than those with high GPR182^+^ PSC scores (HR = 0.62, *p* = 0.007), indicating the prognostic value of GPR182^+^ PSCs in CRC (Fig. 5A). PD-1 and CTLA-4 are immune checkpoints that facilitate tumor escape from the immune system, for which checkpoint-targeted immunotherapy brings encouraging efficacy, especially in patients with resistant or recurring disease. By analyzing TCGA COAD cohort, we found that GPR182^+^ PSC score positively correlated with PD-L1 (*p* = 0.04, r = 0.61) and CTLA4 expression (*p* = 0.02, r = 0.42) (Figs. 5B, 5C). This indicates CRC patients who harbor high proportion of GPR182^+^ PSCs might respond better to checkpoint-inhibitor-based immunotherapy. Drug sensitivity analysis using pRRophetic and oncoPredict packages revealed distinct response profiles between CRC patients with high and low GPR182^+^ PSC scores. Based on the CGP 2016 database, patients with high GPR182^+^ PSC scores tended to show increased response rates to AT-7519, AZD8055, and rapamycin. Conversely, patients with low GPR182^+^ PSC scores were predicted to be more sensitive to Tipifarnib, XMD13-2, Bosutinib, Midostaurin, AS601245, KU-55933, and GDC0449. Analysis of the GDSCv2 database indicated that patients with low GPR182^+^ PSC scores tended to be more responsive to LY2109761, CDK9, temozolomide, AT13148, and luminespib, whereas PF13 was predicted to exhibit promising efficacy for patients with high GPR182^+^ PSC scores. Additionally, FAP samples had higher proportions of GPR182^+^ cells than normal controls, ranging from 0% to 0.12% (Fig. S6A). However, unlike COAD samples, FAP patients and FAP-derived organoids showed no positive correlations between GPR182^+^ PSCs and PD-L1 or CTLA-4 levels (Figs. S6B, S6C, S7B, S7C). Furthermore, FAP patients or organoids with high GPR182^+^ PSC scores displayed distinct sensitivities with specific chemotherapeutics (Figs. S6D and S7D), such as dasatinib and metformin, indicating GPR182^+^ PSC has limited predictive efficacy of across different FAP datasets. Taken together, our findings illustrate the potential applications of GPR182^+^ PSCs in CRC prognosis stratification, treatment modalities, and therapeutic development.

## Discussion

FAP is a familial disorder caused by APC mutation. Patients with FAP have an almost 100% risk of developing colorectal cancer during their lifetime (Esplin et al., 2024; Zaffaroni et al., 2024). To date, neither colectomy nor available drugs can cure FAP (Li et al., 2020). Although APC inactivation is known to drive FAP pathogenesis, further studies are needed to clarify the dynamic pathological features of FAP and to identify novel therapeutic targets and agents. In the present study, we outlined the heterogeneous profiles of polyp epithelial cells in FAP using single-cell RNA sequencing data, established a model to classify FAP patients according to different progression stages, identified a special tumor-priming cell population, GPR182^+^ PSCs, and explored the potential application of these cells in predicting prognosis, immunotherapy responses, and drug sensitivities.

By using gene signatures derived from single-cell RNA sequencing, we were able to discriminate between FAP samples at different stages efficiently (S1–S4) using only transcriptomic data. This approach expands the utility of existing FAP transcriptomic data, as FAP is a rare syndrome and bulk samples are difficult to obtain (Aihara, Kumar & Thompson, 2014). Of note, metabolic reprogramming progressively increased from stages S1 to S4, which is consistent with improved HIF, EMT, and TGF-β gene set scores (Fig. 2). All of these pathway alterations are involved in the transition from benign polyps to high-grade adenomas and ultimately to malignant carcinomas. Although metabolic reprogramming is a recognized hallmark of cancer, its significance in precancerous polyps of FAP patients remains unclear. In line with our findings, Esplin et al. (2024) also discovered metabolic changes in precancerous lesions from FAP patients. Furthermore, the activation of hypoxia-related genes in S2–S4 subpopulations indicates that hypoxia plays an important role during the transition from precancerous lesions to invasive adenocarcinomas. This is consistent with the known role of hypoxia in promoting angiogenesis and metastasis in aggressive tumors (Yuan et al., 2024). Hypoxia in precancerous lesions may result from long-term effects of APC mutation, since APC has been shown to antagonize hypoxia- inducible factors (HIFs) like HIF-1α (Newton et al., 2010).

The epithelial cell subset we identified, GPR182^+^ PSCs, deserves particular attention due to its extensive interaction with immune cells, specifically CD8^+^ T cells and M2 macrophages. Initially characterized as an orphan G-protein coupled receptor, recent research has highlighted GPR182’s role in removing chemokines like CXCL9 and CXCL10, which are predominantly produced by macrophages in many tumors (House et al., 2020; Le Mercier et al., 2021; Torphy et al., 2022). Since these ligands (CXCL9, CXCL10, and CXCL11) and their receptor, CXCR3, play an essential role in guiding effector T cells into tumors (House et al., 2020), a high abundance of GPR182^+^ PSCs may significantly impair the efficacy of immune checkpoint therapies. Moreover, GPR182 depletion has been correlated with increased CD8^+^ T cell infiltration in the TME (Torphy et al., 2022), directly linking GPR182^+^ PSCs to immune suppression. Importantly, our findings show a strong positive correlation between the abundance of GPR182^+^ PSCs and the expression of immune checkpoint molecules, PD-L1 and CTLA-4, in COAD samples, also highlighting a regulatory role of these cells in maintaining an immunosuppressive niche within the TME. Similarly, up-regulated PD-L1 on the surface of other colorectal stem-like populations was also observed (Zhao et al., 2024). The immunosuppressive TME shaped by GPR182^+^ PSCs could selectively support tumor subclones capable of evading immune destruction, thereby contributing to the heterogeneity in FAP patients. Another possible source of heterogeneity driven by GPR182^+^ PSCs might relate to their ability to hijack and distort normal stem-cell traits, such as plasticity and differentiation capacity. GPR182^+^ PSCs may guide the transition of stem cells from a quiescent state to an invasive phenotype or promote their differentiation into subpopulations with varied maturity and functionality.

The above-mentioned crosstalk between GPR182^+^ PSCs and immunity might distinguish them from other established stem markers. For instance, CD133^+^/CD44^+^ cancer stem cells inhibit T cell activities through high-expression of membrane-bound IL-4 (Zhao et al., 2024). Six1^+^ stem-like cells secret high levels of CSF-1, CCL2/5 and VEGF, thereby recruiting tumor-associated macrophages that foster immunosuppression (Xu et al., 2017). These crosstalk patterns are different from those of GPR182^+^ PSCs because of the chemokine-scavenging capacity of GPR182. Moreover, GPR182^+^ PSCs also differ from other stem-like populations in terms of localizations, proliferation and differentiation capabilities, and signaling pathways. For instance, LGR5^+^ stem cells residing at the crypt base exhibit high proliferative activity driven by Wnt/β-catenin signaling (Zhao et al., 2024); SMOC2^+^ stem cells, which are also localized at the crypt bottom, drive a more mesenchymal like phenotype by integrin-linked kinase (ILK) signaling (Shvab et al., 2016); Bmi1^+^ populations situated at the +4 positions represent quiescent ISCs that function weakly to homeostatic regeneration but confer striking resistance to radiation injury *via* epigenetic regulation (Yan et al., 2012). By contrast, GPR182^+^ PSCs have been reported to act as a “brake” on mitotic activity in normal mucosae through ERK signals (Kechele et al., 2017), yet they promote CSC growth by facilitating G1-G2 transition in our study. This discrepancy may arise from the different models used between studies. The animal models used by Kechele et al. (2017) captured a systemic phenotype within an intact microenvironment, whereas our experiment specifically revealed the effect of GPR182 over-expression in epithelial cancer stem cells. Overall, further investigations are needed to clarify the function of GPR182^+^ PSCs.

The role of GPR182^+^ PSCs in drug response prediction for CRC patients is encouraging and promising. Midostaurin (Rydapt) was first approved for the treatment of acute myeloid leukemia in 2017 (Kim, 2017) and has been recently reported to induce colon cancer cell apoptosis and enhance the efficacy of anti-PD-1 therapy in animal models (Lai et al., 2022). Our prediction furthermore indicates midostaurin has better efficacy in CRC patients with low abundance of GPR182^+^ PSCs. Additionally, rapamycin, an mTOR inhibitor, was predicted to be effective for CRC patients harboring high GPR182^+^ PSC scores. Interestingly, rapamycin was shown to be capable of slowing polyp progression and was well-tolerated in FAP patients, whose efficacy has been under investigation in phase II clinical trials (Johnson & Disis, 2025). Recent finding by Habib, Mamistvalov & Ben-Yosef (2025) revealed that rapamycin was able to restore APC-mutated colon organoid differentiation with high mTOR activities, indicating personalized use of rapamycin in FAP patients is needed.

There are also several limitations in this study. Our results are primarily derived from bioinformatic de-convolution of single-cell transcriptomic and bulk RNA-seq data, and were only validated using *in vitro* experiments. These approaches cannot fully capture the dynamic interaction between GPR182^+^ polyp stem cells, stromal fibroblasts, and immune subsets *in vivo*. Additionally, the number of FAP specimens with single-cell transcriptomic data is still small. Larger, multi-center cohorts are required to confirm these findings. Future work should generate lineage-specific GPR182-knockout or -over-expression models and test whether genetic or pharmacologic GPR182 blockade reduces polyp number, size and intra-tumoral heterogeneity.

## Conclusions

In summary, we characterized the heterogeneity of the polyp epithelium in FAP and identified a key epithelial subpopulation, GPR182^+^ PSCs, using machine-learning methods. We demonstrated that GPR182^+^ PSCs could drive tumor initiation, interact with immune populations to foster an immunosuppressive microenvironment. Furthermore, this stem-cell population shows promise for predicting patient prognosis and therapeutic responses. These findings provide novel insights into FAP pathology and therapeutic development.

## Supplemental Information

10.7717/peerj.20704/supp-1 Supplemental Information 1 Supplemental Figures and Table.

## Abbreviations

FAP: familial adenomatous polyposis
EMT: epithelial-mesenchymal transition
GPR182^+^ PSCs: G protein-coupled receptor 182-positive polyp stem cells
APC: adenomatous polyposis coli
CRC: colorectal cancer
SCS: single-cell sequencing
TME: therapy on the immune-tumor microenvironment
RPKM: per million mapped reads
PCA: principal component analysis
PCs: principal components
PAM: Partitioning Around Medoids
CDF: cumulative- distribution-function
GSVA: Gene Set Variation Analysis
ssGSEA: single-sample gene set enrichment analysis
SVM: support vector machine
XGBoost: extreme gradient boosting
RF: random forest
ROC: receiver-operating-characteristic
IF: immunofluorescence
CCK-8: cell counting kit-8
OD: optical density
PI: propidium iodide
CSC: Cancer stem cell sphere
CGP: Cancer Genome Project
GDSC: Genomics of Drug Sensitivity in Cancer
APOBEC3B: apolipoprotein B mRNA editing enzyme catalytic subunit 3B
IGLL5: immunoglobulin lambda-like polypeptide 5
CYP3A4: Cytochrome P450 family 3 subfamily A member 4
CCL20: C-C Motif Chemokine Ligand 20
RGMB: RGM Dotando Beta
PTPRO: protein tyrosine phosphatase receptor type O
LGR5: Leucine-Rich Repeat-Containing G Protein -Coupled Receptor 5
EPHB2: Ephrin Receptor B2
SMOC2: SPARC Related Modular Calcium Binding Protein 2
NORAD: Non-coding RNA Activated By DNA Damage
RPL13: Ribosomal Protein L13
HIFs: hypoxia-inducible factors
ILK: integrin-linked kinase

## Additional Information and Declarations

### Data Availability

The following information was supplied regarding data availability:

The raw measurements are available in the Supplemental Files.

The code is available at Zenodo Ruoyu, W. (2025). Identification of a GPR182-postive stem cell population that drives polyp progression in familial adenomatous polyposis [Data set]. Zenodo. https://doi.org/10.5281/zenodo.17037611

The Flow Cytometry data is available at Zenodo: Ruoyu, W. (2026). Identification of a GPR182-postive stem cell population that drives polyp progression in familial adenomatous polyposis [Data set]. Zenodo. https://doi.org/10.5281/zenodo.18404481

The Tissue experiment raw data is available at Zenodo: Ruoyu, W. (2026). Identification of a GPR182-postive stem cell population that drives polyp progression in familial adenomatous polyposis [Data set]. Zenodo. https://doi.org/10.5281/zenodo.18404438.
