# Supplementary material for: Identification of a GPR182-postive stem cell population that drives polyp progression in familial adenomatous polyposis
Source: PeerJ. 2026 Mar 27;14:e20704. doi: 10.7717/peerj.20704 (PMC13034868; doi:10.7717/peerj.20704)
Supplement: Supplemental Information 1 [file peerj-14-20704-s001.docx]

**Supplementary Figures and Table**


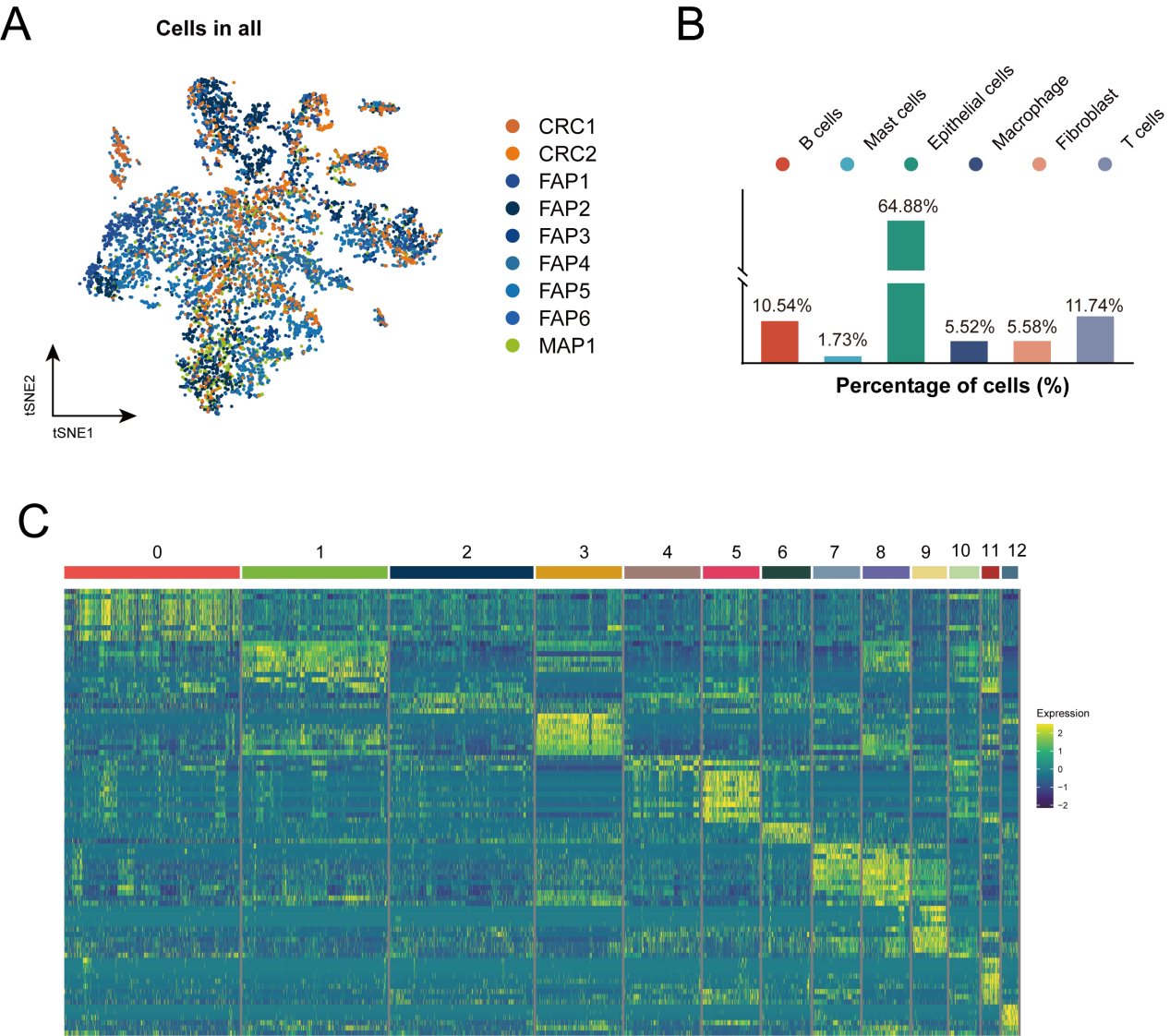


Figure S1. Diverse cell types in FAP delineated by single cell transcriptomic analysis.

1. After data filtering and tSNE analysis, 8086 cells were classified into multiple distinct clusters, including epithelial cell, B cell, mast cell, T cell, macrophage, and fibroblast;

(B) Among these clusters, epithelial cells were the majority, comprising 64.88% of the classified cells from our study;

(C) Epithelial cells were subjected to ClusterTree analysis (the optimal RNN, 0.5), in which 13 clusters with distinct gene signatures were identified.


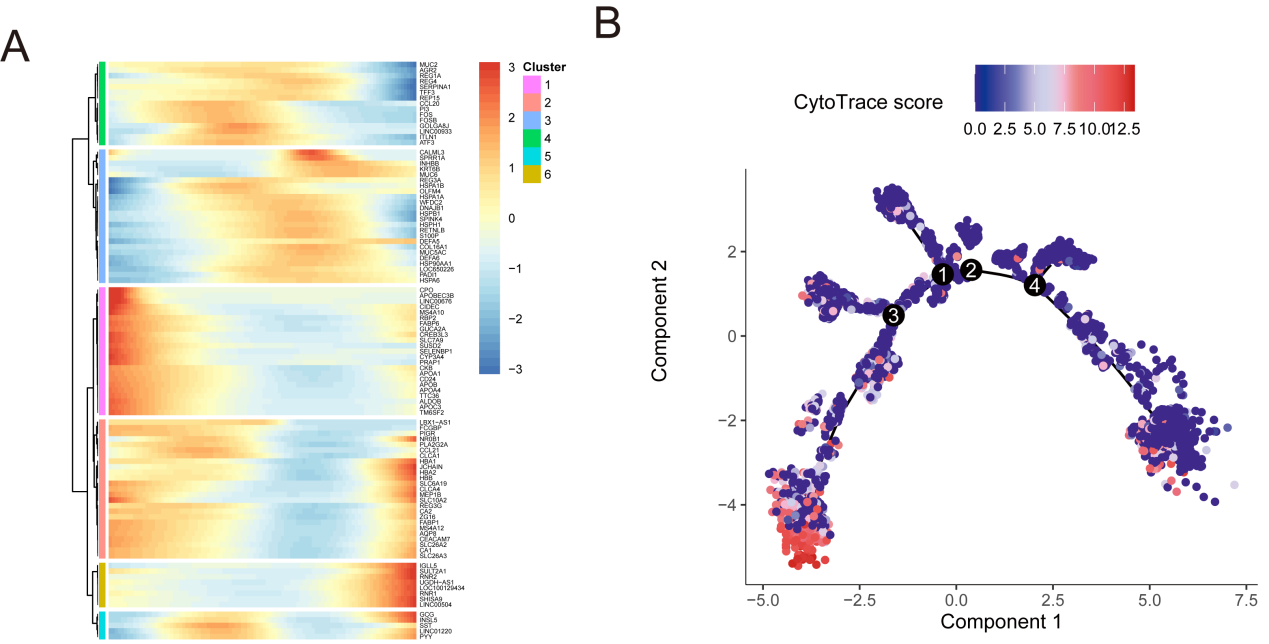


Figure S2. Analysis of single-cell trajectories among 13 epithelial cell subsets.

1. Differentially expressed genes over the Pseudo-time were clustered hierarchically into six profiles. The representative genes were shown;
2. Pseudo-time of CytoTrace score in epithelial cells inferred by Monocle2. Each point corresponds to a single cell.


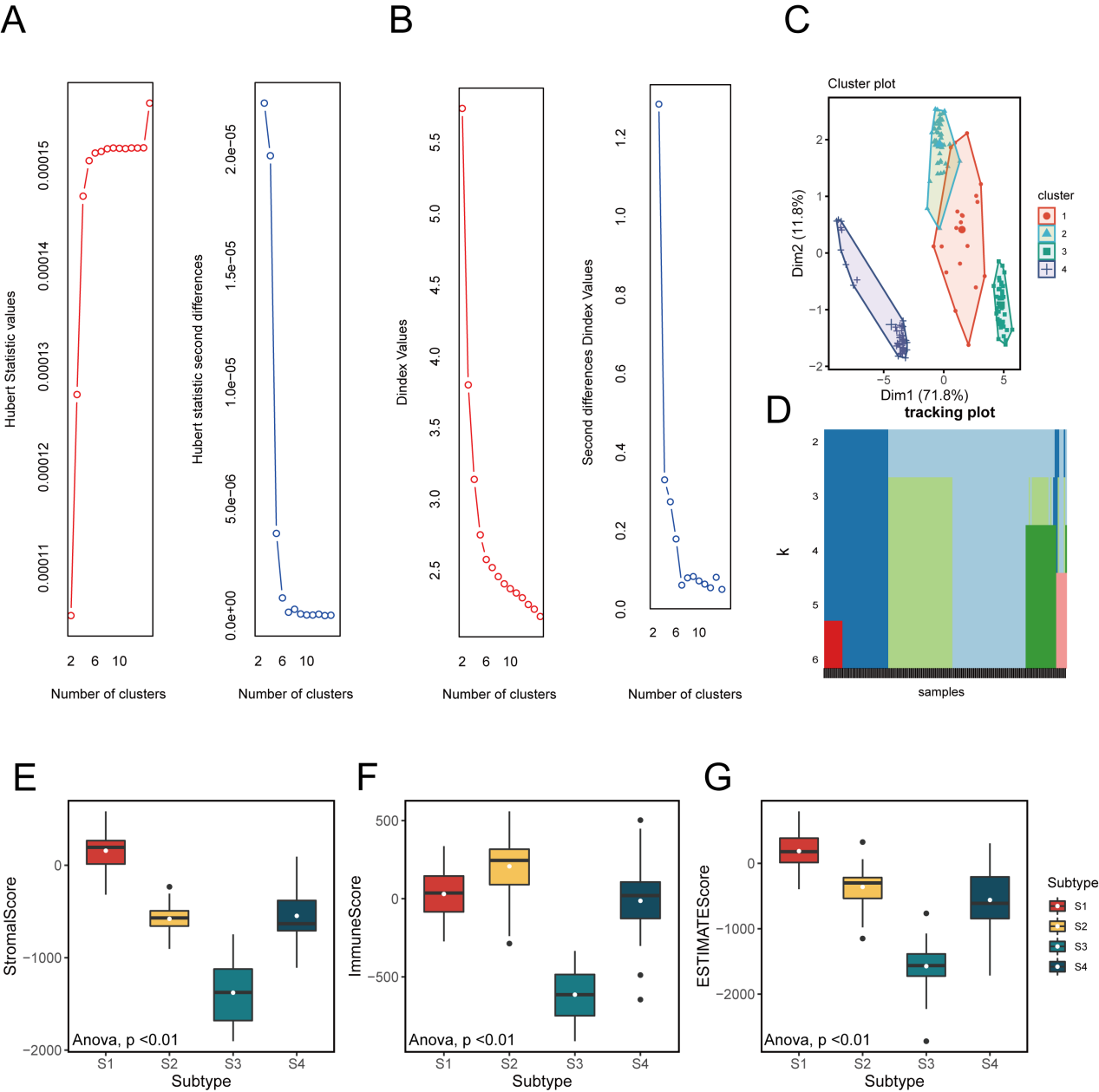


Figure S3. FAP samples can be clustered into 4 subtypes according to transcriptome by gene signatures of 13 epithelial cell subsets.

1. Hubert statistic values and Hubert statistic second difference of different cluster numbers selected in PAM K-mean algorithm;
2. Dindex values and second difference Dindex values of different cluster numbers selected in nbclust algorithm;
3. Dim plot of best cluster number calculated by Within Sum of Squares(WSS);
4. Representative tracking plot of consensus cluster result;
5. Box plot of stromal score in 4 FAP subtypes generated by ESTIMATE R package;
6. Box plot of immune score in 4 FAP subtypes generated by ESTIMATE R package;
7. Box plot of ESTIMATE score in 4 FAP subtypes generated by ESTIMATE R package.


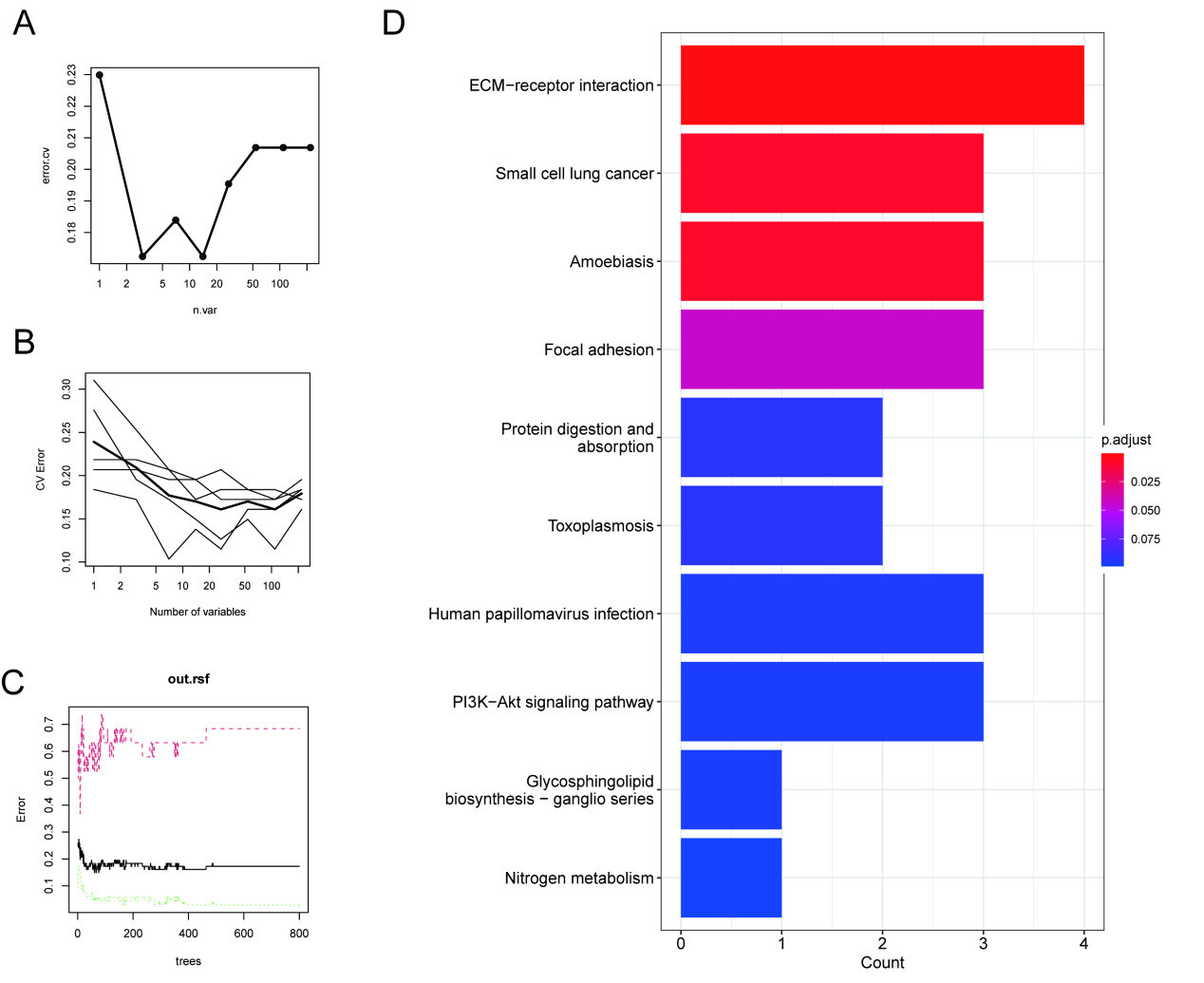


Figure S4. GPR182^+^ polyp stem cells (GPR182^+^ PSCs) were investigated by using machine learning.

1. Error rate of cross-validation in different numbers of variables selected in SVM algorithm;
2. Error rate of cross-validation in different numbers of variables selected in XGBoost algorithm;
3. Error rate of cross-validation in different numbers of trees established in random forest algorithm;
4. Representative enriched GO terms in differentially expressed genes between GPR182^+^ PSCs and other subsets, respectively.


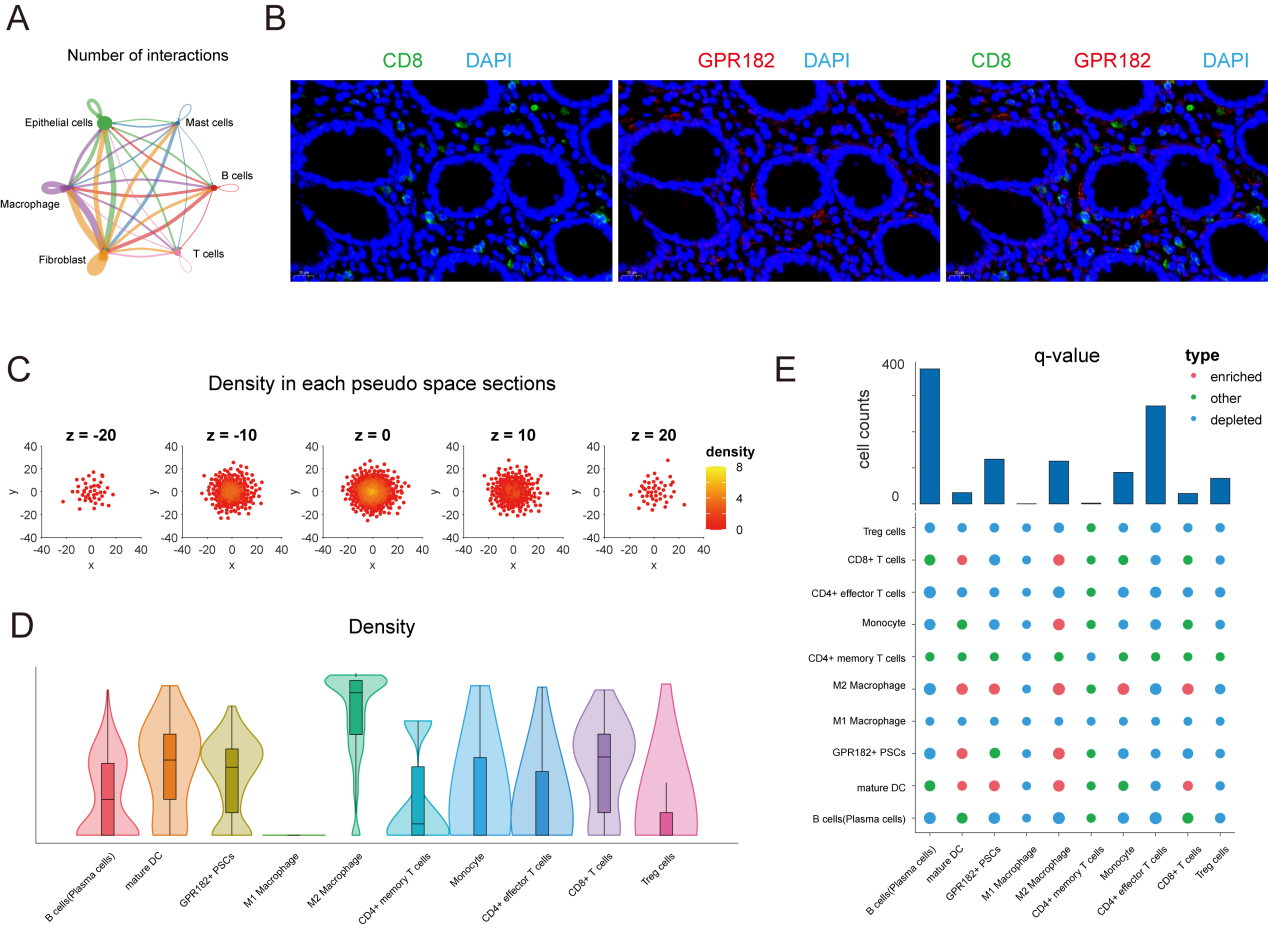


Figure S5. (A) Cell-to-cell communication between FAP epithelial cells and different cell types in TME; (B) Immunofluorescence images revealed GPR182+ cells resided next to CD8a+ T cells. (C) Cross-section views (z = -20, -10, 0, 10, 20) of cell densities in the reconstructed spatial organization of FAP adenoma cells; (D) Violin plot showing the compactness of different cell types; (E) Interaction connections among cell groups and their statistical significance. Enriched, cells from one cell group are enriched in the same region as another cell group (q < 0.05); Depleted: cells from one cell group are depleted in the same region as another cell group (q < 0.05).


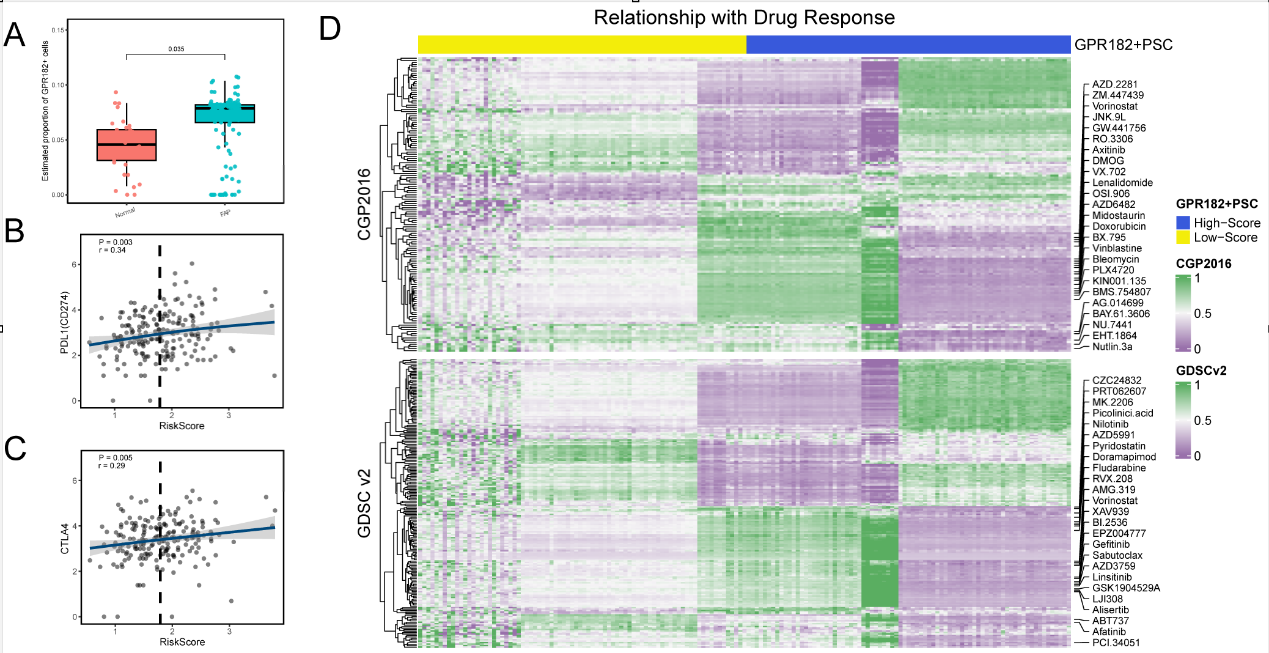


Figure S6. (A) Proportions of GPR182^+^ PSCs within FAP tissues and normal mucosae were compared. (B-C) Correlations between PD-L1, CTLA-4, and proportion of GPR182^+^ PSCs in FAP patients; (D) For FAP patients, the associations between the GPR182^+^ PSC scores and predicted responses to therapeutics from the CGP2016 and GDSCv2 databases, as analyzed by pRRophetic and oncoPredict, were visualized.


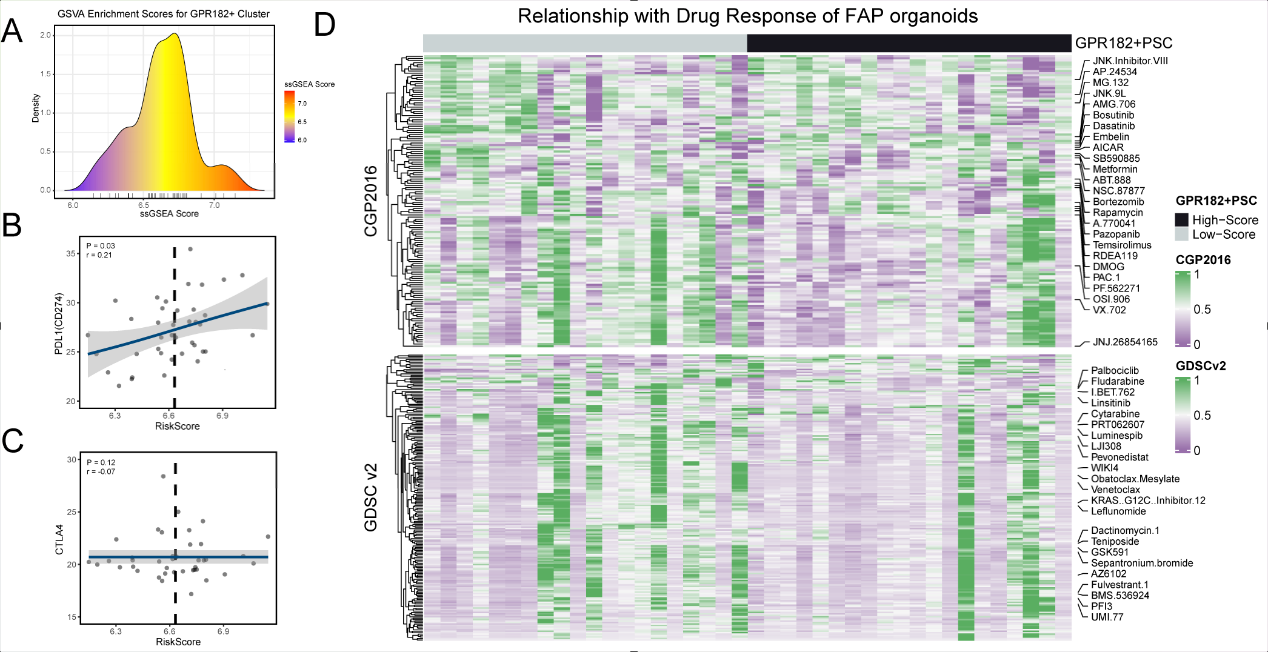


Figure S7. (A) Proportions of GPR182^+^ PSCs within FAP organoids were caculated against ssGSEA scores. (B-C) Correlations between PD-L1, CTLA-4, and proportion of GPR182^+^ PSCs in FAP organoids; (D) For FAP organoids ([GSE207398](https://www.ncbi.nlm.nih.gov/geo/query/acc.cgi?acc=GSE207398); GSE109814), the associations between the GPR182^+^ PSC scores and predicted responses to therapeutics from the CGP2016 and GDSCv2 databases, as analyzed by pRRophetic and oncoPredict, were visualized.

Table S1. Marker genes for each epithelial cell subset.

| gene | p_val | avg_log2FC | pct.1 | pct.2 | p_val_adj | subset |
| --- | --- | --- | --- | --- | --- | --- |
| UGDH-AS1 | 3.54E-81 | 2.412455393 | 0.841 | 0.924 | 7.96E-77 | 0 |
| JCHAIN | 2.28E-78 | 1.656180355 | 0.945 | 0.938 | 5.12E-74 | 0 |
| SHISA9 | 3.69E-65 | 2.348565424 | 0.744 | 0.837 | 8.32E-61 | 0 |
| LINC00504 | 4.97E-56 | 2.125270727 | 0.754 | 0.86 | 1.12E-51 | 0 |
| PARP15 | 7.24E-49 | 1.885635159 | 0.623 | 0.622 | 1.63E-44 | 0 |
| MAB21L3 | 8.30E-39 | 1.947850729 | 0.704 | 0.816 | 1.87E-34 | 0 |
| ABCC9 | 9.89E-38 | 1.945611279 | 0.658 | 0.734 | 2.23E-33 | 0 |
| IGLL5 | 2.61E-32 | 1.914345847 | 0.775 | 0.706 | 5.87E-28 | 0 |
| LOC100129434 | 3.33E-24 | 1.971145967 | 0.646 | 0.768 | 7.50E-20 | 0 |
| OPHN1 | 2.92E-21 | 1.785184906 | 0.616 | 0.719 | 6.58E-17 | 0 |
| TFF3 | 3.08E-215 | 1.946894688 | 1 | 0.976 | 6.94E-211 | 1 |
| FCGBP | 1.67E-166 | 2.399713177 | 0.97 | 0.824 | 3.77E-162 | 1 |
| CLCA1 | 2.89E-152 | 2.281534552 | 0.956 | 0.736 | 6.51E-148 | 1 |
| SERPINA1 | 2.95E-131 | 1.628783824 | 0.912 | 0.631 | 6.64E-127 | 1 |
| ITLN1 | 4.31E-124 | 2.556880723 | 0.821 | 0.565 | 9.70E-120 | 1 |
| REP15 | 1.39E-108 | 1.734576841 | 0.662 | 0.3 | 3.12E-104 | 1 |
| RETNLB | 2.73E-108 | 2.465266681 | 0.414 | 0.1 | 6.16E-104 | 1 |
| ZG16 | 2.51E-39 | 3.169549177 | 0.564 | 0.369 | 5.64E-35 | 1 |
| DEFA6 | 5.40E-35 | 3.365483786 | 0.542 | 0.321 | 1.22E-30 | 1 |
| DEFA5 | 1.22E-17 | 2.927828746 | 0.468 | 0.299 | 2.76E-13 | 1 |
| MALAT1 | 3.46E-92 | 0.763778903 | 1 | 1 | 7.78E-88 | 2 |
| SELENBP1 | 1.86E-60 | 1.152159479 | 0.928 | 0.779 | 4.18E-56 | 2 |
| DAB2 | 9.96E-28 | 1.003450759 | 0.471 | 0.301 | 2.24E-23 | 2 |
| SOX4 | 2.27E-27 | 0.859845126 | 0.858 | 0.75 | 5.11E-23 | 2 |
| SRSF11 | 7.46E-25 | 0.772117758 | 0.809 | 0.717 | 1.68E-20 | 2 |
| CCDC88B | 1.92E-17 | 0.865370803 | 0.549 | 0.446 | 4.32E-13 | 2 |
| DUOX2 | 3.62E-17 | 0.96768448 | 0.533 | 0.427 | 8.15E-13 | 2 |
| PNN | 1.90E-15 | 0.789794473 | 0.672 | 0.597 | 4.29E-11 | 2 |
| EPB41L2 | 3.93E-11 | 0.778846933 | 0.57 | 0.514 | 8.85E-07 | 2 |
| GOLIM4 | 2.18E-10 | 0.828318598 | 0.669 | 0.623 | 4.91E-06 | 2 |
| MUC5AC | 0 | 3.627716856 | 0.897 | 0.054 | 0 | 3 |
| MSLN | 0 | 2.141707581 | 0.777 | 0.069 | 0 | 3 |
| TFF1 | 4.17E-214 | 2.422430875 | 0.923 | 0.265 | 9.40E-210 | 3 |
| COL16A1 | 6.67E-182 | 2.817871671 | 0.894 | 0.299 | 1.50E-177 | 3 |
| REG4 | 9.78E-173 | 2.73107427 | 0.989 | 0.472 | 2.20E-168 | 3 |
| TPM2 | 7.60E-161 | 2.152530165 | 0.769 | 0.226 | 1.71E-156 | 3 |
| SPINK4 | 1.13E-158 | 2.847400987 | 0.989 | 0.773 | 2.54E-154 | 3 |
| S100P | 7.92E-137 | 1.92891782 | 0.992 | 0.596 | 1.78E-132 | 3 |
| MUC2 | 6.93E-116 | 1.73718235 | 1 | 0.871 | 1.56E-111 | 3 |
| CRIP1 | 9.21E-94 | 1.533175842 | 0.942 | 0.663 | 2.07E-89 | 3 |
| ADH1C | 3.11E-112 | 1.77541247 | 0.847 | 0.347 | 7.01E-108 | 4 |
| CD9 | 2.52E-97 | 1.348826384 | 0.997 | 0.777 | 5.67E-93 | 4 |
| MGST1 | 2.39E-91 | 1.322165734 | 0.949 | 0.577 | 5.38E-87 | 4 |
| MGST3 | 2.18E-76 | 1.323206887 | 0.928 | 0.562 | 4.91E-72 | 4 |
| SRI | 3.69E-72 | 1.297708259 | 0.97 | 0.677 | 8.31E-68 | 4 |
| HMGCS2 | 3.28E-57 | 1.314978248 | 0.892 | 0.604 | 7.38E-53 | 4 |
| EDN1 | 1.42E-53 | 1.339647203 | 0.745 | 0.354 | 3.20E-49 | 4 |
| CA2 | 7.12E-42 | 1.668819266 | 0.757 | 0.426 | 1.60E-37 | 4 |
| OLFM4 | 7.36E-34 | 1.81735597 | 0.955 | 0.72 | 1.66E-29 | 4 |
| CA1 | 3.23E-25 | 1.437233557 | 0.529 | 0.251 | 7.27E-21 | 4 |
| MS4A12 | 1.69E-215 | 2.577292102 | 0.835 | 0.129 | 3.81E-211 | 5 |
| CA4 | 6.94E-201 | 2.602774995 | 0.69 | 0.083 | 1.56E-196 | 5 |
| SLC26A3 | 4.87E-194 | 3.096575311 | 0.948 | 0.219 | 1.10E-189 | 5 |
| CLCA4 | 1.25E-188 | 2.477038209 | 0.569 | 0.052 | 2.81E-184 | 5 |
| CEACAM7 | 3.83E-174 | 3.481667919 | 0.911 | 0.237 | 8.61E-170 | 5 |
| CA1 | 3.50E-160 | 2.594144874 | 0.915 | 0.232 | 7.88E-156 | 5 |
| SLC26A2 | 8.96E-145 | 3.563983842 | 0.956 | 0.38 | 2.02E-140 | 5 |
| AQP8 | 1.16E-138 | 2.908128909 | 0.556 | 0.076 | 2.61E-134 | 5 |
| FABP1 | 1.57E-125 | 3.053665648 | 0.996 | 0.634 | 3.53E-121 | 5 |
| GUCA2A | 6.52E-106 | 2.512473339 | 0.677 | 0.163 | 1.47E-101 | 5 |
| MKI67 | 2.43E-183 | 2.12961291 | 0.873 | 0.149 | 5.47E-179 | 6 |
| TOP2A | 1.25E-160 | 2.116625327 | 0.864 | 0.17 | 2.82E-156 | 6 |
| NUSAP1 | 1.61E-139 | 1.79415396 | 0.77 | 0.152 | 3.63E-135 | 6 |
| CENPF | 2.72E-128 | 2.201351247 | 0.779 | 0.168 | 6.12E-124 | 6 |
| HELLS | 1.56E-117 | 1.716290395 | 0.667 | 0.118 | 3.51E-113 | 6 |
| HMGB2 | 1.00E-92 | 1.952008598 | 0.962 | 0.514 | 2.25E-88 | 6 |
| UBE2C | 7.84E-80 | 1.71600667 | 0.709 | 0.219 | 1.76E-75 | 6 |
| TUBA1B | 1.17E-78 | 1.924779425 | 0.991 | 0.711 | 2.63E-74 | 6 |
| STMN1 | 1.70E-63 | 1.607174411 | 0.84 | 0.42 | 3.83E-59 | 6 |
| HIST1H4C | 2.62E-39 | 1.935627368 | 0.948 | 0.748 | 5.89E-35 | 6 |
| LOC650226 | 0 | 2.632573989 | 0.888 | 0.062 | 0 | 7 |
| HSPA6 | 2.80E-209 | 2.321147804 | 0.888 | 0.118 | 6.30E-205 | 7 |
| EVADR | 2.87E-127 | 1.925292617 | 0.665 | 0.104 | 6.47E-123 | 7 |
| ZFAND2A | 2.37E-107 | 1.86287426 | 0.976 | 0.34 | 5.33E-103 | 7 |
| HSPH1 | 1.36E-91 | 2.288123503 | 0.995 | 0.579 | 3.06E-87 | 7 |
| BAG3 | 6.64E-86 | 1.491085329 | 0.942 | 0.349 | 1.50E-81 | 7 |
| FKBP4 | 7.67E-77 | 1.480690503 | 0.942 | 0.413 | 1.73E-72 | 7 |
| HSPD1 | 2.09E-75 | 1.632153676 | 1 | 0.689 | 4.69E-71 | 7 |
| HSPB1 | 1.27E-73 | 2.055856804 | 1 | 0.786 | 2.86E-69 | 7 |
| DNAJB1 | 2.44E-71 | 1.700229282 | 0.99 | 0.758 | 5.49E-67 | 7 |
| BAG3 | 1.25E-115 | 2.491813628 | 0.942 | 0.349 | 2.82E-111 | 8 |
| ZFAND2A | 7.60E-107 | 2.297944244 | 0.951 | 0.342 | 1.71E-102 | 8 |
| C10orf10 | 4.15E-99 | 2.117018963 | 0.782 | 0.21 | 9.34E-95 | 8 |
| HSPA1B | 1.28E-96 | 2.57909158 | 1 | 0.806 | 2.88E-92 | 8 |
| HSPH1 | 1.49E-93 | 2.348505371 | 0.981 | 0.58 | 3.35E-89 | 8 |
| DNAJB1 | 7.53E-89 | 2.132378423 | 0.99 | 0.758 | 1.70E-84 | 8 |
| HSPB1 | 2.99E-85 | 2.544066763 | 0.99 | 0.787 | 6.74E-81 | 8 |
| HSPA1A | 1.01E-78 | 2.174972617 | 0.99 | 0.815 | 2.28E-74 | 8 |
| WFDC2 | 5.65E-61 | 2.24342782 | 0.937 | 0.56 | 1.27E-56 | 8 |
| LYZ | 5.12E-39 | 2.010447261 | 0.922 | 0.653 | 1.15E-34 | 8 |
| PYY | 0 | 5.419368025 | 0.763 | 0.015 | 0 | 9 |
| INSL5 | 8.84E-295 | 3.191176302 | 0.52 | 0.01 | 1.99E-290 | 9 |
| GCG | 1.36E-294 | 3.34610608 | 0.645 | 0.022 | 3.06E-290 | 9 |
| PCSK1N | 2.61E-120 | 2.056392393 | 0.382 | 0.023 | 5.87E-116 | 9 |
| B3GNT7 | 1.13E-108 | 2.020262604 | 0.849 | 0.187 | 2.55E-104 | 9 |
| PI3 | 1.48E-84 | 3.242546074 | 0.961 | 0.37 | 3.33E-80 | 9 |
| LGALS2 | 6.46E-83 | 1.918874269 | 0.829 | 0.227 | 1.45E-78 | 9 |
| CCL20 | 1.99E-70 | 2.414481204 | 0.993 | 0.604 | 4.47E-66 | 9 |
| CXCL1 | 8.63E-67 | 2.275949073 | 0.947 | 0.466 | 1.94E-62 | 9 |
| CXCL3 | 1.54E-60 | 2.046757591 | 0.941 | 0.501 | 3.46E-56 | 9 |
| DUSP5 | 2.32E-55 | 1.579390881 | 0.855 | 0.358 | 5.22E-51 | 10 |
| JCHAIN | 3.60E-54 | 2.161418604 | 1 | 0.938 | 8.11E-50 | 10 |
| CA2 | 1.29E-53 | 1.508895681 | 0.962 | 0.436 | 2.91E-49 | 10 |
| RGS1 | 1.75E-44 | 1.615016359 | 0.947 | 0.64 | 3.94E-40 | 10 |
| KLF4 | 6.19E-43 | 1.498011217 | 0.977 | 0.72 | 1.39E-38 | 10 |
| TSC22D3 | 1.19E-41 | 1.761137027 | 0.878 | 0.523 | 2.67E-37 | 10 |
| FOS | 6.20E-40 | 1.345244162 | 1 | 0.933 | 1.40E-35 | 10 |
| FOSB | 8.00E-40 | 1.529550805 | 0.992 | 0.814 | 1.80E-35 | 10 |
| BTG2 | 1.17E-33 | 1.209313503 | 0.985 | 0.791 | 2.64E-29 | 10 |
| MCL1 | 1.37E-33 | 1.219637043 | 0.908 | 0.677 | 3.08E-29 | 10 |
| APOA1 | 4.77E-292 | 5.368680093 | 0.974 | 0.037 | 1.07E-287 | 11 |
| APOA4 | 2.61E-267 | 3.323194506 | 0.649 | 0.014 | 5.88E-263 | 11 |
| APOC3 | 1.47E-195 | 3.151239726 | 0.481 | 0.01 | 3.32E-191 | 11 |
| FABP6 | 6.95E-191 | 5.222559471 | 0.935 | 0.062 | 1.56E-186 | 11 |
| ALDOB | 1.04E-127 | 4.54663922 | 0.974 | 0.115 | 2.33E-123 | 11 |
| GSTA1 | 9.53E-81 | 3.160738916 | 0.844 | 0.131 | 2.14E-76 | 11 |
| GUCA2A | 5.04E-66 | 3.234162501 | 0.896 | 0.181 | 1.13E-61 | 11 |
| PRAP1 | 2.19E-39 | 3.965541944 | 0.935 | 0.366 | 4.92E-35 | 11 |
| ANPEP | 6.45E-38 | 3.197443409 | 0.753 | 0.218 | 1.45E-33 | 11 |
| ADIRF | 4.44E-18 | 3.081356633 | 0.909 | 0.528 | 1.00E-13 | 11 |
| MUC6 | 0 | 2.639987301 | 0.746 | 0.009 | 0 | 12 |
| KRT6B | 0 | 2.474378683 | 0.831 | 0.019 | 0 | 12 |
| KRT17 | 6.84E-146 | 3.001928309 | 0.859 | 0.066 | 1.54E-141 | 12 |
| BMP4 | 5.82E-63 | 1.787311915 | 0.746 | 0.123 | 1.31E-58 | 12 |
| PTPRO | 1.00E-47 | 1.868644771 | 0.676 | 0.128 | 2.25E-43 | 12 |
| CEACAM6 | 2.95E-39 | 2.658767171 | 0.958 | 0.475 | 6.63E-35 | 12 |
| SCD | 1.38E-26 | 1.728636629 | 0.69 | 0.228 | 3.11E-22 | 12 |
| IFI6 | 3.92E-21 | 1.66184343 | 0.831 | 0.4 | 8.82E-17 | 12 |
| S100A4 | 2.81E-20 | 1.641191209 | 0.775 | 0.366 | 6.32E-16 | 12 |
| CXCL8 | 3.75E-17 | 1.622624507 | 0.915 | 0.561 | 8.44E-13 | 12 |
